# Process Intensification Approach Using Microreactors for Synthesizing Nanomaterials—A Critical Review

**DOI:** 10.3390/nano11010098

**Published:** 2021-01-04

**Authors:** Vikas Hakke, Shirish Sonawane, Sambandam Anandan, Shriram Sonawane, Muthupandian Ashokkumar

**Affiliations:** 1 Chemical Engineering Department, National Institute of Technology, Warangal 506004, India; hakke.vikas@gmail.com; 2 Department of Chemistry, National Institute Technology, Trichy 620015, India; 3 Nano Research Project Laboratory, Department of Chemical Engineering, Visvesvaraya National Institute of Technology, Nagpur 440010, India; shriramsonawane@gmail.com; 4 School of Chemistry, University of Melbourne, Melbourne, VIC 3010, Australia

**Keywords:** microreactors, nanoparticles, continuous flow, segmented flow, process intensification

## Abstract

Nanomaterials have found many applications due to their unique properties such as high surface-to-volume ratio, density, strength, and many more. This review focuses on the recent developments on the synthesis of nanomaterials using process intensification. The review covers the designing of microreactors, design principles, and fundamental mechanisms involved in process intensification using microreactors for synthesizing nanomaterials. The microfluidics technology operates in continuous mode as well as the segmented flow of gas–liquid combinations. Various examples from the literature are discussed in detail highlighting the advantages and disadvantages of microfluidics technology for nanomaterial synthesis.

## 1. Introduction

### 1.1. Microfluidics Technology for Process Intensification

Conventional reactors have many bottleneck problems such as energy losses (mass as well as heat), high wastages, poor reaction control, and high operational risk. The conversion of reagents depends upon space–time in the reactor and the mixing kinetics inside the reactor. Due to the large quantity, it is difficult to achieve a uniform mixing inside conventional batch reactors leading to dead zones. Many solutions have been proposed after a decade of research such as the modification of the reactor internal surface with baffles but still, the problem of mixing kinetics remains unresolved with batch reactors. All these issues ultimately reduce the yield and quality of the product [1,2].

In recent decades, process intensification has gained importance due to its effectiveness in the utilization of resources available. Maximum conversion, high safety and minimal or optimal energy losses are the primary focus in the intensified design approach. Microfluidics is one of the smart solutions generated by the intensification design approach. A new generation of micro dimension reactors are developed to reduce the overall fabrication cost, size, optimal utilization of space and enhanced safety [3]. The compact size of the reactors induces good mixing conditions which improve the reaction kinetics. In the intensified reactor design approach, one of the dimensions is in the submillimeter range. These new generation reactors are referred to as microstructured devices/reactors, or microreactors [3,4]. The major advantage of microreactor technology is the higher surface-to-volume ratio which can be as high as 100,000 m^2^/m^3^ [5]. The heat and mass transfer rates are synergistically improved due to the small volume and high surface area, which may reduce the reaction time to a few seconds from several hours. The safety associated with the process is enhanced due to the small volume of the reactor.

### 1.2. Nanostructures and Need for Microfluidics Technique in Their Synthesis

Nanomaterials have been used for many centuries, however, the intense interest arrived only in recent decades. A large number of synthetic approaches have been developed; examples include chemical vapor deposition, sputtering, spray pyrolysis and electrochemical methods [6]. Nanoparticles are widely applied in the areas of paint, coatings, electronics, textiles, bioimaging, medicine, and many more [7]. Most of these methodologies have some limitations such as poor control over particle size, size distribution, shape, poor reproducibility, and time-intensive synthetic processes. The main challenge is the production of nanomaterials on a commercial scale with control over particle size, shape and polydispersity. Microreactors offer control over these properties of nanomaterials.

In this review, the fundamental aspects of microreactors and a comparative study of different modes of operation are discussed. The critical review also covers the advantages and disadvantages of microreactor technology for the synthesis of nanoparticles.

## 2. Microstructure Reactors Design Principles

### 2.1. Design Consideration for Nanoparticle Synthesis in Microstructured Reactors

The microstructure reactors, which were initially introduced in processing as capillary-type reactors, have now been extended to small volume reactors such as Corning^®^ Advanced-Flow™ Reactors (AFR), New Delhi, India. The synthesis of nanoparticles can be achieved through many physical as well as chemical processes. The commercially available processes select a top-down approach as it is easier for scale-up. Microfluidics uses a bottom-up method, where the chemical synthesis of the nanoparticles is achieved through the sol–gel or chemical reduction methods. The microreactors provide a controlled environment as Reynolds number is very low in the range of 100 to 500 so solute transport is uniform leading to the generation of the desired size and shape of nanoparticles. Before understanding the design principles of the microreactor, one must understand the synthetic mechanism of nanoparticles from their constituents.

#### Chemical Synthesis of Nanoparticles: Bottom-Up Approach

**Figure 1:**
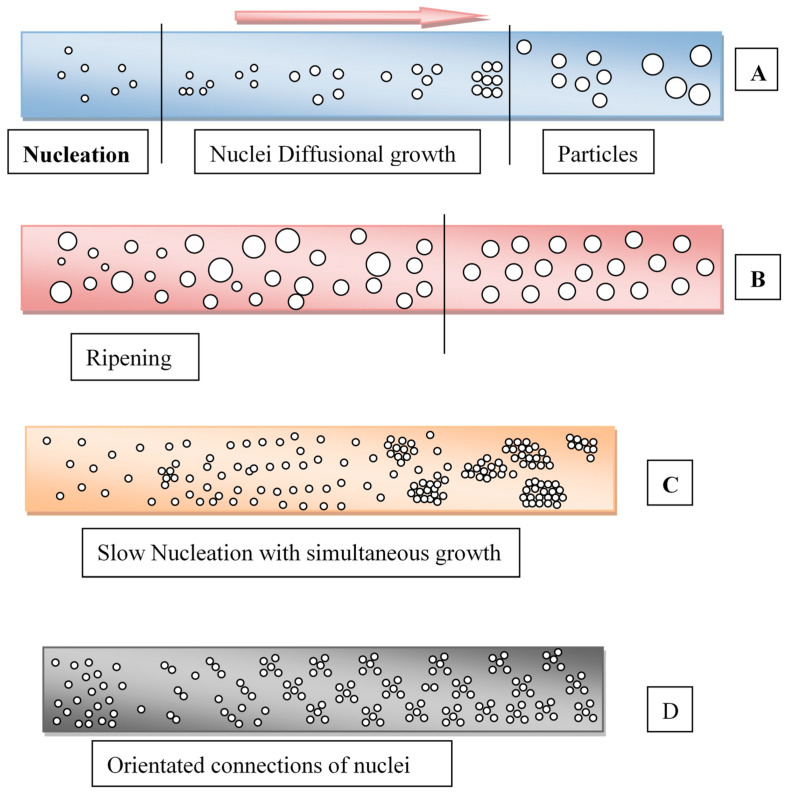
Nuclei growth mechanisms representation (**A**) LaMer approach, (**B**) Ostwald ripening, (**C**) Finke–Watzky and (**D**) coalescence and oriented connections mechanism. The Figure was conceptualized based on the information provided in Ref. [8].

In this section, an overview of the theories of formation and growth of nanoparticles is provided. The chemical synthesis of nanoparticles can be defined by four approaches: (i) LaMer approach, (ii) Ostwald ripening, (iii) Finke–Watzky mechanism and (iv) coalescence (Figure 1). The LaMer model suggests that the diffusion rate of monomers in the solution is responsible for the nanoparticle formation through nucleation and its propagation. This model depends on the concentration gradient-based mechanism. Ostwald ripening is based on the solubility of particles in the solution. The smaller particles, due to their high solubility and surface charges, show higher mobility towards each other. This induces the growth of particles through the re-deposition of smaller nanoparticles. This process is exactly the opposite of digestive ripping. The Finke–Watzky model explains the mechanism in two stages: first is nucleation followed by particle growth. Whereas the theory of coalescence and oriented attachment explains the effect of coalescence on orientation effects in the growth of particles [8].

### 2.2. Microreactors

One-dimensional microscale reactors have their benefits and drawbacks. The nucleation followed by the growth of particles within a constrained environment is the generalized approach for the synthesis of nanoparticles using microreactors. Chemical reactions such as oxidation and reduction of precursors are the most widely used approaches in microreactors. Two or more reactants are brought together in microreactors to achieve a maximum conversion during the reaction. The product with a lower solubility and higher surface energy leads to the formation of nanoparticles, through nucleation and crystal growth. All the processes such as nucleation, growth, precipitation, coagulation and flocculation occur within a fraction of time during the reaction in a microreactor.

The hydrodynamic flow patterns in microchannels have a significant effect on the efficiency of production and properties of nanoparticles. The two-phase flow offers some advantages in comparison with single-phase flows such as increased interfacial area, shortened transfer distance and enhanced mixing, which can reduce mass transfer limitations. The two-phase flow properties in microchannels rely on three parameters: the channel geometry, the properties of both fluids, and the flow conditions. All factors that contribute to the two-phase flows can be described by important dimensionless parameters. As the dimensions of the reactor are reduced to a micrometer scale, the interfacial effects become stronger and significant. The interfaces such as fluid–wall, and fluid–fluid will gain much importance in microchannels. The interaction of the fluids with the wall surface determines the flow pattern in a microchannel. The flow pattern in a microreactor is categorized as ordered and disordered flow patterns depending upon fluid–wall interface interaction. When a continuous phase completely wets the microchannel wall, ordered patterns can be obtained. If the wetting is partial, disordered flow patterns are observed [9,10].

The microreactors are divided into two categories: a segmented flow microreactor, and a continuous flow microreactor. The flow rates of the reactants in the microchannels play a wide role. Khan et al. [11] demonstrated the difference between one-phase and two-phase segmented flow. The macro lumps of the reaction mixture in the microchannel were created by bubbling inert gas in the microchannel along with reactants. The reaction mixture flows in the microchannels as micro pockets as similar to the plug flow reactor. During the synthesis of silica nanoparticles, they reported that when the reactor proceeded with the segmented flow regime, the synthesized particles had a narrow size distribution. They also reported that this effect was due to a well-mixed condition in the microreactor.

#### 2.2.1. Segmented Flows

When two or more immiscible fluid streams are mixed in a microchannel is considered as a segmented flow microreactor. This discrete flow creates different sections in the channels for two or more reactants providing associated space–time for the reaction. Gas–liquid, liquid–liquid and liquid–solid systems can be treated in segmented flow microreactors [12]. Two-phase flow in a microchannel, where one phase as microdroplets flows through the length of the microreactor, has been utilized in various systems. It was first developed as capillary microreactors for drug delivery in droplets as a dispersed phase.

**Figure 2:**
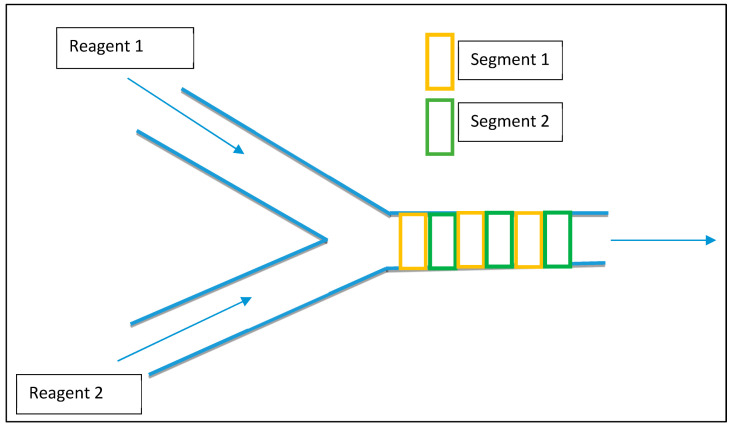
Segmented flow microreactor.

The usual method of generating droplets (emulsion) consists of uneven droplet size and irregular mixing and dispersion. Moreover, the process needs additional energy input for stabilizing emulsion [13,14]. The slug flow microreactor provides an effective solution with minimal energy input for the dispersion of droplets. Figure 2 illustrates a segmented flow in the microreactor. The basic model consists of a Y-junction where the immiscible phases come in contact with each other [15]. The segmental flow in channels depends upon various factors such as initial injection velocities of immiscible fluids, microchannel geometry, and the material of construction (MOC) of the microreactor. The initial flow rates of input streams and the affinity of fluid towards the MOC of microchannels will be helpful for the manipulation of a particular phase in droplets (discrete flow). The balance between immiscible phases and the flow rate of input streams at the Y-Junction of the microreactor will produce the segments in the channel flow. Generally, the phase with a high affinity towards MOC of microchannels produces a continuous phase in the channel, and fluid with lower affinity is converted into a discrete phase as droplets. Often, laminar flows are utilized for efficient contact and mixing for such cases. The shape of the microreactors, such as a winding geometry, may help to control the droplet properties [16]. The geometrical variations in microchannels are significant, as they help to convene three different streams in a confined region (microchannel). In many cases, a centrally mounted orifice is used to create the discrete segmented flow, where the droplet of the immiscible phase will be smaller than that of the orifice opening and the phases are well mixed due to a pressure difference. The orifice opening or any obstacle to the flow will disturb the central fluid stream and this destabilization will be helpful for the formation of segments in the channel [14,17,18]. Surfactants present in the continuous phase can be used for the stabilization of droplets in the channel. When chemical reactions are carried out in these microchannels or droplet-based reactors, the composition at the liquid–liquid interphase changes along the length of the microchannel which leads to highly efficient conversion of reactants in the desired product. That is why microreactor technology is efficient for producing nanoparticles [19]. Other types of continuous microreactors include capillary microreactor, coaxial flow microreactor and micromixing-based microreactor [20,21].

#### 2.2.2. Spinning Disc Processing (SDP)

**Figure 3:**
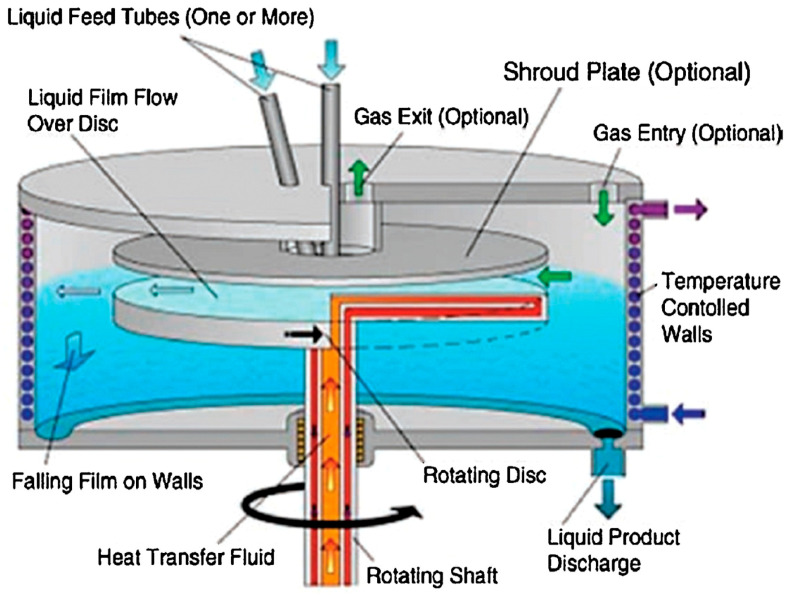
Schematic of a spinning disc processor (SDP) (reprinted [23] with permission from Elsevier, 2017).

Spinning disc processing (SDP) is an important milestone in fluid dynamics research and a landmark in process intensification modernization [22]. The spinning disc processing helps to produce greater control over the size of nanoparticles and for synthesizing new materials that normally cannot be synthesized via other processing techniques, in addition to overcoming the problems associated with the scaling-up process. The key components of SDP include a rotating disc with controllable speed and feed jets located at the center of the disc. SDP generates a very thin fluid film (1–200 μm) on a rapidly rotating disc surface (300–3000 rpm), within which nanoparticle formation occurs (Figure 3) [23]. Several factors (choice of solvent, reducing agent, stabilizer, pH, and temperature) need to be taken into account, while synthesizing metal nanoparticles. The reductant and stabilizer are the most important parameters which will determine the final particle size.

Exploitation of a spinning disc reactor (SDR) to achieve the efficient mixing profile, will depend upon the energy dissipation along the solution. SDRs consist of a high-speed rotating disc, impeller and dynamic seal which has a high power requirement through the rotating pump. The rotating mechanical elements have their own limitations due to friction, wear and tear associated with the metal. The dynamic sealing of the disc at high rotating speed is itself a challenging task. Dynamic sealing can be broken down into rotational movement. Rotational movement describes the seal spinning around a fixed axis while maintaining adequate sealing force in application. Rotary seal designs must take into consideration shaft diameter, shaft rotational speed (RPM), and surface finish of the mating hardware. The actual energy dissipated in the solution, which is utilized for the generation of high shear stress, is much less than that of the impinging-jets microreactor power consumption of auxiliary elements [24,25,26].

#### 2.2.3. Geometrical Variations in Microreactor

Often, reagents flow in the microchannel in the laminar flow region, as a result of which the critical issue of mixing arises. At a lower velocity profile, molecular transport was controlled by molecular diffusion. The geometrical variation such as helical structure may be the solution. The winding geometry of the microchannel is constructed on the Dean vortex, which utilizes the centripetal force when fluid flows in a circular path in microchannels. This additional centripetal force, which is usually directed outward, creates the pair of counter-rotating vortices in the moving fluid. These counter vortices at the inner and outer wall of the microchannels enhance the mixing index of the fluid. The favorable well-mixed conditions in a helical structured microreator are notably found to be capable of synthesis of controlled shaped and size of nanoparticles with efficient conversion. The Dean vortex is generated in the development of the region of high pressure in microchannels which reduces the cost of pumps for the generation of pressure. Roudet et al. [27] explore the advantages of meandering geometry with respect to straight channels. The higher improvement was found in the mass and heat transfer coefficient with the geometrical variation of the microchannel [28,29]. Kockmann et al. [30] found that the symmetrical vertices were built in the straight line microreactors as flow velocity moving towards transient flow regime. At the entrance of mixing, channel vortices increase the mass and heat transfer coefficient [31].

## 3. Nanoparticles Synthesis Using Microreactors

The nanoparticles synthesized with the help of microreactors can be classified into three major categories of inorganic nanoparticles, organic nanoparticles and nanocomposites.

### 3.1. Organic Nanomaterials

Organic nanomaterials are finding significant opportunities in various sectors, specifically in pharmaceutical industries. Nowadays, nanomedicines are being developed and utilized in the diagnosis and treatment of diseases like cardiovascular, cancer, and infectious diseases [32]. The synthesis of organic nanomaterials for medicinal applications was carried out via the production of nanoliposomes in 1965. Nanoliposomes were used as a drug delivery system to deliver various bioactive molecules to targeted cells. However, a major concern in the production of these novel materials was the difficulties in obtaining uniformity and reproducibility in physicochemical properties. Microfluidics and microreactors can offer better control over the shape and size of the organic nanomaterials and thus find their applications for the synthesis of organic nanomaterials. Production of nanoliposomes in microchannels generated liposomes with a mean diameter of around 12 µm with unilamellar morphology [33]. Whereas, the microfluidic crossflow injection method resulted in homogenous nanoliposomes with a size between 200 and 500 nm [34]. These organic nanoparticles show better stability, solubility, and availability at the targeted objectives [35,36,37,38].

Genot et al. [39] have synthesized the organic rubrene nanocrystals with a nonsolvent crystallization process in a 3D hydrodynamic focusing microreactor. They observed an average particle size of around 50–110 nm. They had succeeded in operating the microreactor without any deposition on the inside wall of microchannels, with the help of a focusing ratio. The ratio of side flowrate to the capillary flowrate (focusing ratio), has a great effect on the nucleation rate in the microchannel. The authors reported that as the focusing ratio increases, the average nanocrystal size decreases. With the rise in focusing ratio, the capillary flowrate reduces, which gives adequate time for the nucleation and growth of nuclei in the capillary, and due to side flowrate, the crystalline structures become well mixed in the stream. The quality of mixing is enhanced as the focusing ratio increases which was the major reason for the restricted growth of organic nanocrystals in the process.

The biopolymer nanoparticles have a wide range of applications due to their properties such as appreciable rheological properties, water dispersibility, texture, appearance, and many more. They have applications in the food industry for the encapsulation of bioactive compounds and in the wastewater treatment industry [40]. Recently, Kim et al. [41] utilized a continuous droplet-based microreactor for the synthesis of drug nanoparticles of Itraconazole using an antisolvent precipitation method. They obtained Itraconazole nanoparticles with a droplet-based reactor. The particle size they found showed narrower size distributions in solution. Jose et al. [42] developed a continuous technique for precise and scalable synthesis of 2D metal-organic frameworks. They studied the precipitation kinetics in the solution flowing through the microchannel. They develop the continuous process framework for the organic material-based nanoparticle synthesis. They also reported that the conversion rate was higher than that of the batch reactor by five orders of magnitude through the process. Jaouhari et al. [43] proposed the synthesis of organic nanoparticles in a microreactor with the additional effect of the supercritical antisolvent process. They used tetrahydrofuran for the stabilization and carbon dioxide as supercritical fluid to get nanoparticles of tetraphenylethylene in a range of 10 nm scale. The authors successfully implemented a supercritical antisolvent method with a microreactor to achieve the nanoscale organic material. The CO_2_ and tetrahydrofuran binary system showed high solubility variation for tetraphenylethylene. The CO_2_ worked as an antisolvent for tetra-phenylethylene, as the concentration of CO_2_ increased in the system the nucleation of tetra-phenylethylene increased. The growth of nuclei was restricted due to high-pressure microreactors. Kaeko et al. [44] utilized a thin-film reactor to prepare the curcumin with a size distribution from 50 to 100 nm. This microreactor consisted of two disks, and a nanoparticle thin layer was formed in between the two rotating concentric disks. The authors found that the crystallization mechanism was limited in the precipitation of a solid when escaping from the thin layer of the film. However, the addition of continuous crystallization of organic material helped to enhance the yield. They also studied the effect of parameters such as rotation speed, time, temperature and pressure effect on the continuous process.

### 3.2. Inorganic Nanomaterials

**Figure 4:**
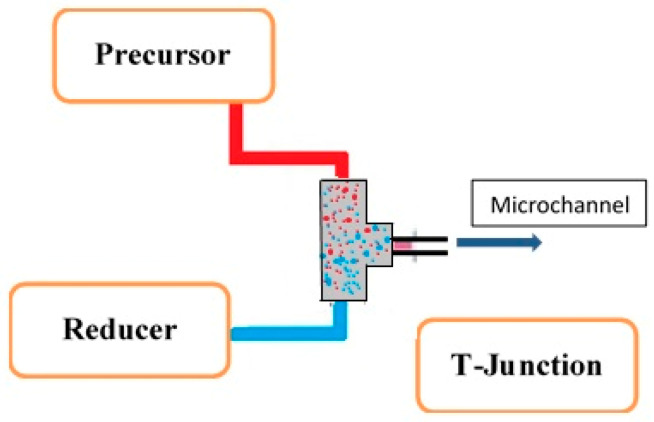
Schematic representation of T-junction.

Inorganic nanomaterials have several applications; this was due to their selective electronic structures. This electronic structure is most favorable for electron transfer. The metal nanoparticle synthesis follows typical stages such as nucleation, propagation of nuclei to crystal, and growth of crystals through flocculation, followed by agglomeration. The reduction of precursor, simultaneous redox reaction within the solution and solvent extraction are the common synthetic procedures for the nanoparticles. The reduction of precursors in microchannels was effective for the synthesis of inorganic metal nanoparticles. During the synthesis of nanoparticles, factors such as inlet flow rates, precursor concentration, reaction residence time, reaction temperature, surface properties all affect the size and morphology of nanoparticles. In general, after the nucleation process, the microchannel facilitates the growth of nuclei up to the desired size, and the agglomeration was avoided by dispersing the nanoparticles in the stabilizing solution or buffer solution as per the requirement. For the homogeneous nanoparticles, a rapid nucleation process and controlled growth of nuclei are needed. Yen et al. [45] synthesized CdSe nanocrystals by using the continuous flow microcapillary reactor. Due to its effective bandgap capacity to tune for a wide visible region, this semiconductor nanocrystalline structure of CdSe was in great attention. They also reported that residence time distribution, inherent nucleation, and progress of growth for the nuclei play an important role in the size restriction on the synthesized nanocrystals of CdSe. Song et al. [46] reported nanoparticles of cobalt synthesis by using microfluidics technology. The residence time of the reactant in the reactor is very much important, as it directly affects reaction time, flowrates, and geometry of the reactor. With the help of variation, Song et al. [46] synthesized the different crystal morphology for cobalt nanoparticles. Most of the inorganic nanoparticles synthesized in the microreactor were reported through the bottom-up approach. The precursors utilized for the reaction are injected in the microreactor with a specific flowrate which combines in the microchannels. The effective combination of a junction in the microchannels is an important parameter. The mixing junction in microchannels decides the synthesis rate, morphology, and size of the ultimately formed nanoparticle. Sharada et al. [47] carried out the reduction of a palladium precursor (PdCl_2_) with the help of NaBH_4_ to obtain Pd nanoparticles. They used a simple capillary microreactor and injected PdCl_2_ and NaBH_4_ through the T-junction at the inlet of the capillary illustrated in Figure 4.

**Figure 5:**
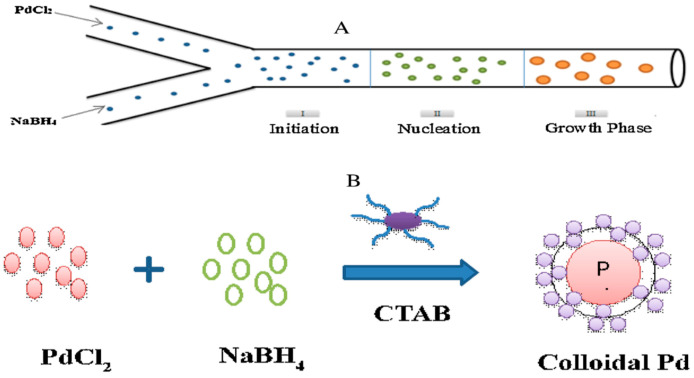
(**A**) Mechanism of palladium nanoparticle formation in a microreactor. (**B**) Representation of reaction scheme for the formation of metallic Pd nanoparticles in the presence of stabilizing agent CTAB (reprinted with permission from Elsevier [47], 2016).

The fixed morphology of cubic shaped Pd nanoparticles was synthesized using the reduction in the microchannels. Figure 5 shows the experimental flow diagram for the synthesis of Pd nanoparticles. The precursor concentration was taken as a limiting component, and its concentration showed an impact on the size of nanoparticles. As the precursor’s concentration was low, the yield of nanoparticles was also low. The growth progress of the synthesized nanocrystals in the microchannels was restricted due to the low availability of Pd in the solution and crystals generated showed a smaller size range of about 5 nm. The reaction time also affected the particle size, they reported that as the flow rate increased, the average particle size of Pd nanoparticles also increased. This is due to the presence of unreacted precursors at the time of the crystal growth process, which resulted in an increase in the crystal size.

Gioria et al. [48] synthesized palladium nanoparticles (PdNPs) by effective utilization of green reagents like glucose and starch. They performed the reaction in two different configurations for the comparison. One through the conventional beaker batch method and the second with continuous microreactor technology. They reported that microreactors led to smaller PdNPs with a monodispersed size distribution and higher turnover frequency for the synthesized particle. They also reported that the stability of nanoparticles was higher. The catalytic response of both materials was tested on the reduction of 4-nitrophenol. The smaller PdNPs formed in the continuous flow reactor showed a higher catalytic activity due to the high catalytic surface area of the nanoparticles.

On a similar note, Wagner and Kohler [49] studied the synthesis of Au nanoparticles using a microfluidics technology through a bottom-up approach. They used HAuCl_4_ and ascorbic acid in microchannels. They also explored the effect of many parameters, such as the pH, flow rate, and reagent concentrations were screened to tune the nanoparticle properties. As the pH of the reaction solution increased, the average particle size decreased. They also reported the effect of the diameter of channels on particle size distribution. They reported that as the diameter of the channels was altered, the morphology of synthesized nanoparticles was influenced due to changes in mass transfer. A plant extract was used as a reducing agent in the preparation of Ag nanoparticles in a tubular microreactor by Jolhe et al. [50]. They concluded that the presence of ascorbic acid in the bio-extract of R. Sativus was responsible for the reduction of a silver precursor. They also varied the diameter of the microreactor to understand the effect on the structures of particles generated. From the results, they inferred that a larger flow area promoted better mass transfer which resulted in a better growth of nuclei.

Gioria et al. [51] employed starch and glucose as green reagents to obtain Pd nanoparticles. They utilized the reducing capacity of glucose and starch was used as a stabilizing agent. The size of Pd nanoparticles synthesized in continuous-flow microreactors was found to be around 100 nm, and showed higher surface area and efficient catalytic activity.

Yu et al. [52] exploited an impinging stream microreactor for the synthesis of nanosized α-Al_2_O_3_ powders. The properties of α-Al_2_O_3_ nanoparticles prepared by a conventional precipitation process were compared with those prepared by the microchannel method. They found that the microchannel method produced homogeneous α-Al_2_O_3_ nanoparticles due to the rapid nucleation rate.

The exploitation of the coaxial flow reactor for the synthesis of silver nanoparticles was demonstrated by Baber et al. [53]. They found that the size of the microchannel had a significant effect on the size and distribution of nanoparticles. They also studied the effect of residence time and concentration of surfactant and the reaction kinetics. These authors discovered that as the flow rate decreased, the size of nanoparticles increased due to rapid nucleation at the inner and outer interface of two streams. At a lower flow rate, the residence time of reactants in the microreactor is high due to which agglomeration process can be observed and nanoparticles synthesized were larger in size. However, at a high flow rate, the size of nanoparticles was low at the cost of lower conversion. Xu et al. [54] synthesized Ni nanoparticles in aqueous solutions using hydrazine as a reducing agent in a continuous flow microchannel. The length of the reactor was varied to provide the required resident time for the reactants that resulted in the desired size range and morphology of the Ni nanoparticles. One of the important operating parameters to get the consistent performance of microreactors is temperature. Garcia-Manrique et al. [55] showed the effect of temperature on continuous microreactors performance. He et al. [56] synthesized the metal-organic framework on the glass fiber membrane in the microreactor.

Most often, these nanoparticles are supported by carrier/support materials. These supporting materials ensure the effective and economical utilization of nanoparticles as a catalyst. Hornung et al. [57] proposed the coating of Pd nanoparticles and Li et al. [58] proposed coating of TiO_2_ nanoparticles on the inner surface of microchannels to ensure the effective utilization of the catalyst in microchannels. Feng et al. [59] designed a microchannel for the effective utilization of Pd nanocatalyst. In their design, they coated the inner layer of the microchannel with polydopamine layer and Pd nanoparticles deposits alternately. They found that the Pd nanocatalyst utility, dispersibility, and availability in multiphase reactions were enhanced and a higher process intensification was achieved. These microchannels were found to efficiently convert the reactants, finds maximum yield and selectivity during the reaction.

Turkevich et al. [60] were the first to produce gold nanoparticles of a certain controlled size by reduction of tetrachloroauric acid with citric acid or sodium citrate in boiling water using SDP. The citrate was oxidized to dicarboxyacetone which underwent further reaction to form a reducing species, acetone. The main advantage of SDP is that it led to the formation of gold nanoparticles in just a fraction of a second. By pumping 1 mM AuCl_4_^−^ and 10 mM sodium citrate into the SDP at a rate of 0.5 mL/s by maintaining the disc speed (300 rpm) and temperature (150 °C), dark red gold particles with particle sizes between 5 and 20 nm were generated. Similarly, through the SDP approach, star-shaped gold nanoparticles were prepared using ascorbic acid as a reductant at ambient temperatures. Another green reductant, glucose, was also used to produce gold, however, the solution needed an alkaline condition for glucose ring-opening by α-proton abstraction for the conversion of glucose to gluconic acid by the AuCl_4_^−^ salt [61]. It is interesting to note that just boiling off the solution is sufficient for the generation of silver nanoparticles by SPD [22,23]. Silver nanoparticles were produced by pumping silver nitrate and glucose in the ratio of 1:5 through the feeds by varying the disc speed from 300 to 3000 rpm at the disc temperature (120 °C). The reaction for the reduction of silver cations by glucose (glucose is oxidized to gluconic acid) is as follows:

Ag^+^ + C_6_H_12_O_6_ + 2OH^−^ → Ag^0^ + C_6_H_12_O_7_ + H_2_O (1)

Here, glucose played both as a surfactant and a reducing agent. In continuation, Iyer et al. [22] prepared silver nanoparticles of a varied size range, 5–200 nm, by carefully adjusting the concentration of the feed and as well as regulating the disc speed in the presence of reductant ascorbic acid and soluble starch as a stabilizing agent. The effect of reactant concentration on the morphology of synthesized silver nanoparticles was also reported [26]. They reported that as the concentration of silver nitrate was increased, particle size increased and with lower disc speed, the particles generated showed a narrower size distribution, possibly because the reaction occurred in the collection trough and was not completed in the SDP. Besides the above synthetic strategies, the implementation of an environmentally friendly approach may be attractive where hydrogen gas is used as a reductant. Since the standard reduction potential of hydrogen is 0 V, it does not reduce silver salts to silver metal and to reduce it needs a potential of −1.8 V (E° (Ag^+^/Ag^0^)). Hence, researchers tried various approaches to overcome this obstacle and one of the approaches is the saturation of sodium polyphosphate solution with hydrogen, which causes binding of Ag^+^ ions to the polyphosphate chain (acting as nucleating sites) for the formation of Ag metal.

In this context, Hartlieb et al. [62] tried silver nanoparticle growth using the phosphonated calixarene as a template, surfactant, and stabilizer along with hydrogen gas (reductant). They showed the formation of red-colored Ag nanoparticles in the presence of the phosphonated calixarene (pH 9) due to the presence of phenolic groups in the lower rim of calixarene that get oxidized. Further, the advantage of this approach is that water-soluble calixarenes offer scope for systematically tailoring the size of nanoparticles. TEM results illustrated that the average particle diameter can be changed between 2 and 15 nm upon changing the concentration of the phosphonated calixarene, and besides, the particles look crystalline with the typical face-centered cubic structure.

**Figure 6:**
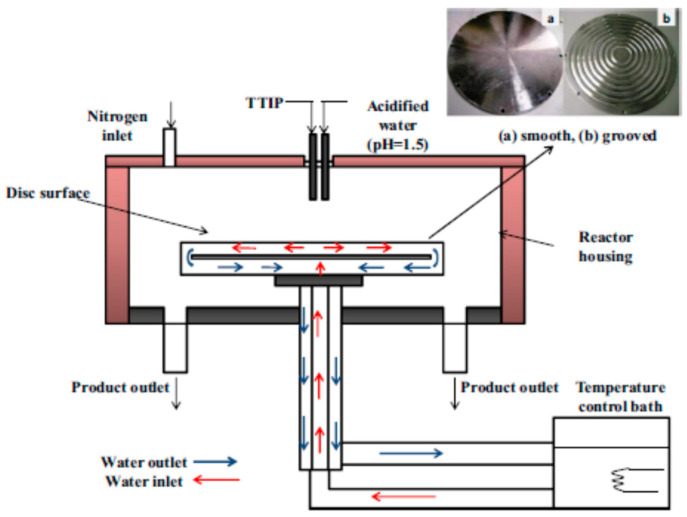
Scheme of set up used for TiO_2_ precipitation experiments (**a**) smooth surface disc, (**b**) grooved surface disc (reprinted with permission of Elsevier [63], 2014).

**Figure 7:**
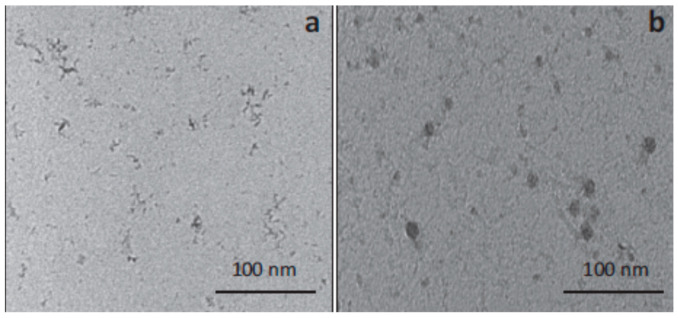
TEM images of TiO_2_ at 1200 rpm, ratio of 12 and flowrate of 10.8 mL/s (**a**) grooved disc, (**b**) smooth disc (reprinted with permission from Elsevier [63], 2014).

Furthermore, an SDR has salient characteristics towards the production of semiconductor nanoparticles due to the uniform and rapid micromixing environment that occurs when two liquid streams are contacted on the rotating surface. The main novelty of SDRs is the generation of well-defined crystals in terms of their size, morphology, and purity under a wide range of disc speeds and flow rates. In this context, TiO_2_ nanoparticles of the desired size were synthesized by an SDR (Figure 6 shows set-up used for TiO_2_ precipitation experiments) via the solgel route at a relatively low processing temperature (50 °C) by hydrolysis of titanium tetra isopropoxide (TTIP) with acidified water (pH 1.5) [63]. The property of the disc surface (grooved and a smooth disc) is found to affect the particle size (grooved disc results in smaller and narrower particle size distribution as compared to a smooth disc) (Figure 7).

**Figure 8:**
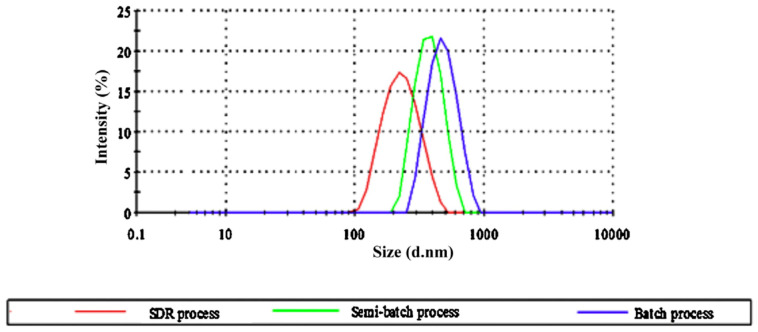
Particle size distribution profile for a spinning disc reactor (SDR), batch and semi-batch process at solvent–non-solvent (S-NS) ratio 1:4, curcumin concentration in the solvent 0.5 g L^−1^ and PVP concentration in non-solvent 1 g L^−1^, for SDR disc (18 cm groove) speed 1500 rpm and conventional reactor impeller speed 1000 rpm (reprinted with permission of Elsevier [65], 2014).

Hartlieb et al. [64] investigated the formation of ZnO nanoparticles through SDP. They dissolved zinc nitrate in an ethanol solution along with 0.1 wt % polyvinylpyrrolidone (PVP) as one feed whereas the second feed contained the desired base in ethanol solvent. These feed solutions were then fed onto the surface of a 10 cm grooved or smooth spinning disc at a certain flow rate, rotating speed, and temperature. Afterward, they determined the size of the particles from the absorption spectra (Figure 8, Table 1) which clearly illustrated that the variation of SDP parameters strongly influenced the rate of nucleation. Temperature also influenced the amount of ZnO formed. From the SDP results, it may be concluded that very small nanoparticles can be generated compared to other sol–gel approaches; besides, the particle size and polydispersity were found to increase with an increase in disc temperature.

Nanosizing via SDP is one of the attractive approaches used to alleviate poor drug dissolution problems (for example, preparation of curcumin—a yellow polyphenol nanoparticle). In comparison to other top-down and bottom-up processes, SDP acts in reducing particle size, surface area and eventually increases the solubility and bioavailability of the poorly soluble drug component. Khan et al. [65] prepared curcumin nanoparticles by SDR mixing and compared the results with batch and semi-batch processes by fixing operating parameters such as curcumin concentration 0.5 g L^−1^ in the solvent (S), PVP concentration 1 g L^−1^ in non-solvent (NS) (S-NS ratio 1:4). The results illustrated that curcumin particles prepared via an SDR were smaller in size (235 nm) compared to batch (451 nm) and semi-batch (356 nm) processes (Figure 8). Further, they studied the dissolution rate of curcumin nanoparticles prepared via the SDR method which showed a higher water dissolution rate compared to that of pure curcumin due to the higher surface area generated upon nanosizing. This may be due to improved mixing at microscale level in the SDR which results in a smaller particle size with narrow particle size distribution (PSD) of precipitated curcumin nanoparticles whereas sluggish mixing of particles in the batch or semi-batch process leads to agglomeration of particles.

The impinging-jets microreactor is attractive and has recently become popular for the synthesis of inorganic nanomaterials. The extremely high energy streams when passing through the closed conduct of flow area in microscale (microchannel) induce the high level of mixing in the microreactor. The density of kinetic energy associated with the reacting stream often remains constant and creates a well-mixed environment in a small space which is favorable for the nucleation and controlled growth of crystals [66]. The synthesis of nanocrystalline BiFeO_3_ [67], rhabdophane-structured LaPO_4_ [68], nanocrystalline TiO_2_ [69,70] by using an impinging-jets microreactor has been reported in the literature.

### 3.3. Nanocomposite Materials

The synthesis of nanocomposites of two different states of the same materials or if two different individual materials are more attractive. The simultaneous reaction of reduction and oxidation, electronic configuration benefit of two different states of materials such as oxidized state of copper (CuO) and Cu metal composition shows higher semiconductive properties together. Such materials composition in nanoscale shows greater advantages concerning their singular applications. Xu et al. [71] reported the successful synthesis of Cu-CuO nanocomposite in a microreactor. They partially oxidized the portion of Cu nanoparticles in the controlled environment of microchannels. They followed a two-stage synthesis process, in the first stage they reduce the precursor of Cu to get nanoparticles of Cu in a desired spherical shape and minimal size. In the second stage, the synthesized nanoparticles of Cu are oxidized in suspension to get CuO nanoparticles. The product of partial oxidation consists of unreacted Cu nanoparticles as well as reacted CuO nanoparticles. Cu-CuO nanocomposite shows higher semiconductor properties. On the other hand, Knauer et al. [72] exploited the controlled surroundings of microchannels for the simultaneous reduction of two different precursors at one reaction mixture. They found that synthesized nanoparticles had an affinity towards each other and formed a composite structure with van der Waals forces. They used ascorbic acid for the reduction of precursors of Au and Ag simultaneously in the microchannel. The reduction of HAuCl_4_ and AgNO_3_ in a semi-segmented flow microreactor produced double-layered nanocomposite of Au/Ag/Au. Abou-Hassan et al. [73], on a similar path, synthesized magnetic and fluorescent γ-Fe_2_O_3_@SiO_2_ core/shell nanoparticles. Whereas Strab et al. [74] successfully synthesized nanostructured Co_3_O_4_@SiO_2_ particles in a laminar flow reactor in batch operations. However, the particle size distribution was broader due to the laminar regime of flow in a microchannel, but they succeeded in obtaining core-shell nanostructures. The segmented flow pattern showed maximum mixing in the channel, further phase separation interfaces provided a high site for the reaction. Due to higher agitation and well-mixed condition in segmented flow microreactors they produce nanoparticles with narrow size [75]. Khan et al. [36] attempted to develop the shell coating without the second nucleation by manipulating reactions in a multistage microfluidic system. They demonstrated the synthesis of core silica nanoparticles coated with titania layer in continuous flow microchannels. Xu et al. [76] synthesized polyimide precursors in the microreactor through the solution polymerization process. The nanocomposite of polyimide was reported to produce in remarkably less time (20 min) as compared to several hours of the conventional process.

### 3.4. Quantum Dots Production Using Microreactors

The nanoparticles of semiconductive materials show enhanced properties when excited under UV light irradiation. In recent years, quantum dots are getting much more important due to their properties such as electron transmission efficiency, tunable emission, high absorption coefficient, and many more. They have many applications specifically in electronic sensing, solar surfaces, light illuminating applications. Kikker et al. [77] synthesized CdSe and CdTe nanoparticles in a continuous flow microreactor. They found that process to be safer as compared with the batch process, as the operating temperature for the microreactor was 160 °C. They synthesized different sizes of the quantum dots with the variation of residence time distribution (RTD) for the flow reactor. Schejn et al. [78] demonstrated the synthesis of ZnO quantum dots in a microreactor, whereas, Rao et al. [79] synthesized carbon dots with a microreactor. They found the process of synthesis of particles with narrow distribution was easy in a microreactor as compared with conventional batch processes. The synthesized quantum dots show high stability and narrow size distribution which makes them photoluminescent. Similarly, Tang et al. [80], used a microreactor for the synthesis of nitrogen-doped carbon quantum dots. They found that with the nitrogen doping, there is an enhancement in quantum yield. The molecular condensation of nitrogen on the formed core of carbon dots was the reported mechanism by Tang et al. [80]; they reported an increase in quantum yield by 84.1% for the nitrogen doping. Alonsoa et al. [81] synthesized carbon dots with a ceramic microreactor. The design of the microreactor was such that, it was an integrated low temperature confined ceramic microreactor. They use the hydrothermal method to generate the carbon dots at high-temperature and pressure without finding difficulties with the operating conditions. The synthesis of carbon dots with high rates shows substantial efficient stability and high quantum yield. The authors reported that the quantum yield of the dots was dependent on the flow pattern in the microchannel. LiFePO_4_ quantum dots of ultrafine size were synthesized in a microreactor by Wang et al. [82]. They proposed a successful synthesis of LiFePO_4_ with two forms of carbon materials as one with an amorphous phase and another with the conductive graphite phase. Similarly, Jeong et al. [83] synthesized CdSe quantum dots with a modified microreactor. They propose the automated and continuous microreactor design for the synthesis.

### 3.5. Challenges Related to Use Microreactor for Nanoparticle Production

At present, researchers use many types of microreactors for the synthesis of different nanomaterials, which has made it difficult to categorize them. The geometric variations sometimes suitable for the specific application concerning reaction conditions, required size, and shape. However, the small-scale microreactors need to integrate or combine in numbers to meet the large-scale requirement from the industry. The compatible mechanisms of operations need to develop to operate integrated chips for continuous and consistent products. Often, the throughput of the microreactors is inconsistent due to variation in operating conditions. The effects of variations of operating conditions such as reagent flow rates, operating pressure, initial concentration, and the geometry of the channel all alter characteristics of the synthesized nanoparticles. The optimum operating conditions for operating a microreactor are important.

## 4. Particle Size Control Aspects of Microreactor during Synthesis of Nanoparticles

**Figure 9:**
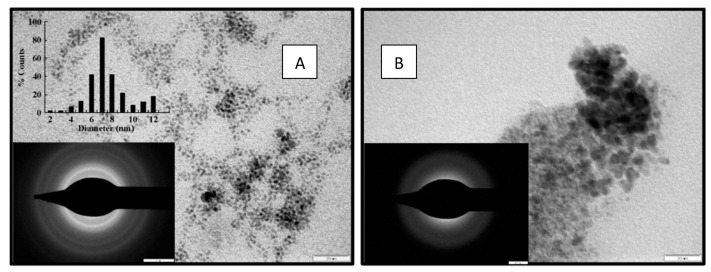
Effect of flow rate in continuous flow microreactor on the nanoparticle size and crystallinity as observed by TEM and Selected Area Electron Diffraction (SAED). (**A**) Flow rate= 14 μL/s and (**B**) flow rate = 28 μL/s (image scale: both TEM 20 nm, both SAED 5 nm) (reprinted with permission with Elsevier [85], 2016).

The time of reaction, operating temperature and pressure, the residence time of reactant in the microchannels, flow pattern/rates in the microchannels, and many more must be controlled. The desired shape and morphology of the nanoparticles have more importance in the application of materials. The conventional batch processes do not have proper mixing, uniform concentration, they do not effectively utilize the reaction sites and many more drawbacks are associated with them; one of the reasons behind that is the size of the operation. In the microreactor, the size and quantity of reactants to be handled were lower so that other operating conditions could achieve the level of maximum ideality to give the expected results. The well-mixed condition in the microchannel, controlled temperature uniform along the radius of the reactor, allows the microreactor to operate near to ideality and generates the maximum of desired products. Patil et al. [84] reported the effect of concentration and flow rates of precursor and reducer in the microchannels on the synthesized nanoparticles. They observed a variation in concentration and flow rates that changed the particle size distribution and morphology of synthesized nanoparticles. They used two surfactants for the synthesis of silver in microchannel and found a difference in the size distribution of nanoparticles. This variation of size distribution was due to the inherent changes in nucleation pattern and controlled growth mechanism of nanoparticles in the suspension. The increasing concentration of surfactants in the reaction mixture shows broader size distribution due to the additional agglomeration effect in the microchannels. In the same line, Suryawanshi et al. [85] studied the effect of surfactant on the particle size distribution in a microreactor. They varied the flow rate, the ratio of precursor to the reducer to the microchannel inlet, the operating temperature of the microreactor, and noted the significant difference in the synthesized nanoparticles of platinum. Figure 9 shows the HRTEM image of synthesized platinum nanoparticles. Lin et al. [86] have also observed similar results which they attributed to changes in solute viscosity at high flow rates.

**Figure 10:**
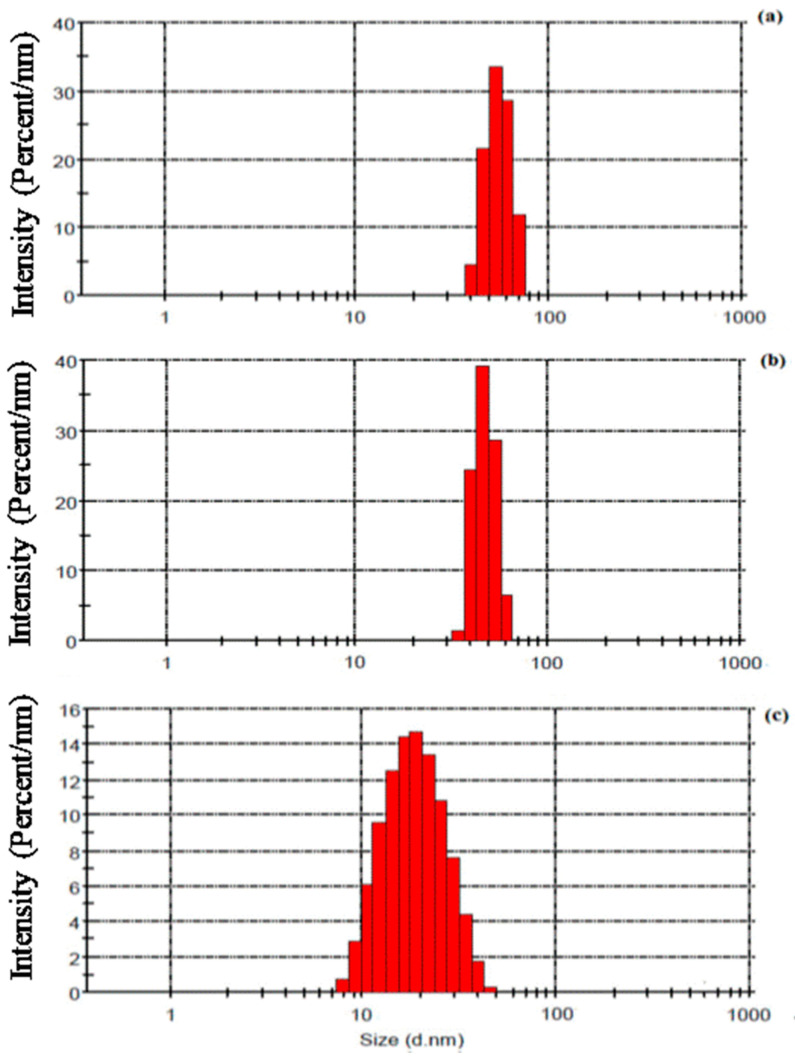
Particle size distribution (PSD) of catalysts (Pt-Mo/C) with different metal (Pt and Mo) loading of (**a**) 40 wt%, (**b**) 30 wt% and (**c**) 20 wt% (adapted from [87], Publisher, 2019).

The inset histogram shows the size distribution of particles synthesized at a flow rate of 14 μL/s [85]. The effect of metal loading in the microreactor has a meaningful effect on the synthesized nanoparticles. Pillai et al. [87] studied the metal loading effect on the synthesis of Pt-Mo/C nanoparticles in a continuous flow milli-channel reactor. They found that as the loading of metal in the microchannel increases, due to flocculation followed by coagulation in growth propagation of crystals particle size of synthesized nanoparticles was enhanced. They vary the metal loading from 20 to 40% and found the size distribution getting broader as shown in Figure 10. The controlled nucleation and the growth mechanism of nanoparticles in the microchannels were dependent upon the concentration of precursor and residence time of reactants in the microreactor. As the residence time of reactants increases in the microchannels, the flocculation in the synthesized nanocrystalline structures was observed due to inherent intermolecular forces and broader size distribution observed [88]. Pillai et al. [87] performed all experiments by maintaining constant residence time; the variation in the particle size of synthesized nanoparticles was predominantly due to the concentration variation of metal loading in the microchannels. The variation in the intermolecular forces at the metastable zone of nucleation growth by the presence of an additional concentration of metals may be the driving cause for the variation in particle size. Similar results were observed by Antolini [89] and Yarlagadda et al. [90].

Peng-Fei Xu et al. [91] reported the geometrical shape of microchannels showed a significant effect on the characteristics of the synthesized nanoparticles. They demonstrated the production of monodispersed sulfur nanoparticles by using two types of mixing junctions one was Y-shaped and another was a T-shape mixing junction followed by the microchannel. The mixing effect in the Y-shaped junction was found to be much better than the T-shaped junction. On a similar line, Huang et al. [92] studied the effect of reactants with the material of construction of microreactors during nanoparticle synthesis. They used a capillary continuous flow microreactor for the synthesis of gold nanoparticles; different materials were used for the construction of the capillary such as polytetrafluoroethylene, fluorinated ethylene propylene, polyetheretherketone, fused silica, and observed the effect of material interaction during the nucleation and crystal growth mechanism. They also reported a surface-to-volume ratio variation study, average residence time, and temperature variation in the synthesis of gold nanoparticles. The surface-to-volume ratio was zero for the slug flow in the microchannels and synthesized nanoparticles were in a narrow zone. They proposed a hypothesis that the surface roughness of the wall of microchannels may provide higher sites for the nucleation and progressive growth of nanocrystals. Nagamine [93] attempted to improve the functionality of the microreactor by using TiO_2_/Ti, as a material of construction. Adamo et al. [94] proposed the cotton threads-based microreactors, for the synthesis of gold nanoparticles. They demonstrated the design of cotton-based microchannels by using 3D printing technology and utilized the same for the synthesis of Au nanoparticles.

## 5. Summary

The drawbacks of the conventional batch process for the synthesis of nanoparticles can be overcome using microreactors. Microreactors provide a continuous, efficient, and safer solution for the synthesis of the desired size, shape, morphology, and composition of nanoparticles. The different structures of microreactors are mainly classified based on the flow pattern of the reaction mixture in the microchannel. The segmented flow or multiphase flow in the microchannel shows efficient results over single-phase flow. The laminar single-phase continuous flow microreactor shows a broader size distribution whereas the multiphase, segmented flow microreactor shows a narrower size distribution of nanoparticles. Microreactors provide a controlled reaction environment in a microchannel, due to which nanocomposites showing core-shell composition can also be successfully synthesized. They have a significant impact on operating conditions and as-prepared nanoparticles. The control aspect of microreactors can be resolved by controlling these parameters during the operation. Overall, microfluidics technology provides an efficient solution over the inefficient utilization of energy during the synthesis of nanomaterials in conventional methods.

## Figures and Tables

**Table 1:** Size and polydispersity for Zn:NaOH:PVP 10 1:1:0.05 wt % (reprinted with permission of Elsevier [64], 2014).

	25 °C Grooved Disk		80 °C Grooved Disk		25 °C Smooth Disk	
Speed (rpm)	Band Gap (eV)	Particle Size (nm)	Band Gap (eV)	Particle Size (nm)	Band Gap (eV)	Particle Size (nm)
500	3.90	2.1 ± 16.2%	3.77	2.5 ± 19.6%	3.95	2 ± 16.9%
1000	4.09	1.75 ± 13.8%	4.00	1.9 ± 18.2%	4.20	1.6 ± 13%
2000	4.20	1.6 ± 13.8%	4.00	1.9 ± 13.5%	4.34	1.45 ± 10.9%
3000	4.29	1.5 ± 11%	4.00	1.9 ± 12.6%	4.49	1.3 ± 10%

## Data Availability

Not applicable.

## Abbreviations

AFR	Advanced-Flow™ Reactor
MOC	Material of Construction
SDP	Spinning Disc Processing
PdCl_2_	Palladium precursor
CTAB	Hexadecyltrimethylammonium bromide
PdNPs	Palladium nanoparticles
TTIP	Titanium tetra isopropoxide
NS	Non-solvent
PSD	Particle Size Distribution
SDR	Spinning disc reactor
CO_2_	Carbon dioxide
NaBH_4_	Sodium borohydride
Pd	Palladium
HAuCl_4_	Chloroauric acid
Al_2_O_3_	Aluminium oxide
TiO_2_	Titanium oxide
AuCl_4_^−^	Tetrachloroaurate ion
C_6_H_12_O_6_	Glucose
C_6_H_12_O_7_	Gluconic acid
ZnO	Zinc oxide
PVP	Polyvinylpyrrolidone
CuO	Cupric oxide
AgNO_3_	Silver nitrate
Co_3_O_4_	Cobalt tetraoxide
SiO_2_	Silicon dioxide
Fe_2_O_3_	Ferric oxide
CdSe	Cadmium selenide
CdTe	Cadmium telluride
RTD	Residence Time Distribution
LiFePO_4_	Lithium iron phosphate
